# A Multicentre, Randomised, Controlled Trial of a Combined Clinical Treatment for First-Episode Psychosis

**DOI:** 10.3390/ijerph18147239

**Published:** 2021-07-06

**Authors:** Itxaso González-Ortega, Patricia Vega, Enrique Echeburúa, Susana Alberich, Jessica Fernández-Sevillano, Sara Barbeito, Vicent Balanzá-Martínez, Eduard Vieta, Esther Lorente-Rovira, Ana Luengo, Ester Cerrillo, José Manuel Crespo, Carlos Matute, Ana González-Pinto

**Affiliations:** 1 Centre for Biomedical Research in the Mental Health Network (CIBERSAM), 28029 Madrid, Spain; patricia.vegaperez@osakdidetza.eus (P.V.); enrique.echeburua@ehu.eus (E.E.); susana.alberichmesa@osakidetza.eus (S.A.); jessica.fernandezsevillano@osakidetza.eus (J.F.-S.); sara.barbeito@unir.net (S.B.); vicente.balanza@uv.es (V.B.-M.); EVIETA@clinic.cat (E.V.); esterlorente@hotmail.com (E.L.-R.); jmcrespo@bellvitgehospital.cat (J.M.C.); anamaria.gonzalez-pintoarrillaga@osakidetza.eus (A.G.-P.); 2 Bioaraba Research Institute, Department of Psychiatry, Araba University Hospital, 01004 Vitoria, Spain; 3 Department of Personality, Assessment and Psychological Treatment, National University of Distance Education (UNED), 01008 Vitoria, Spain; 4 Department of Neurosciences, University of the Basque Country, 48940 Leioa, Spain; 5 Department of Personality, Assessment and Psychological Treatment, University of the Basque Country, Biodonostia, 20018 San Sebastian, Spain; 6 Department of Fundamental Mathematics, National University of Distance Education (UNED), 01008 Vitoria, Spain; 7 Department of Psychology, International University of La Rioja (UNIR), 26006 Logroño, Spain; 8 Teaching Unit of Psychiatry, Department of Medicine, University of Valencia Medical School, 46010 Valencia, Spain; 9 Department of Psychiatry, Hospital Clinic, Institute of Neuroscience, University of Barcelona, IDIBAPS, 08036 Barcelona, Spain; 10 Department of Psychiatry, Clinic University Hospital of Valencia, INCLIVA, 46010 Valencia, Spain; analuenva@hotmail.com; 11 Department of Psychiatry, Bellvitge University Hospital, IDIBELL, 08907 Barcelona, Spain; ecerrial@gmail.com; 12 Achucarro Basque Centre for Neurosciences, University of the Basque Country, 48940 Leioa, Spain; carlos.matute@ehu.eus; 13 Centre for Biomedical Research in Neurodegenerative Diseases (CIBERNED), 48940 Leioa, Spain

**Keywords:** first-episode psychosis, early intervention, treatment, outcome, randomised controlled trial

## Abstract

Introduction: There is evidence that early intervention contributes to improving the prognosis and course of first-episode psychosis (FEP). However, further randomised treatment clinical trials are needed. Objectives: The aim of this study was to compare the efficacy of a combined clinical treatment involving Cognitive Behavioural Therapy (CBT) as an adjunctive to treatment-as-usual (TAU) (CBT+TAU) versus TAU alone for FEP. Patients and methods: In this multicentre, single-blind, randomised controlled trial, 177 participants were randomly allocated to either CBT+TAU or TAU. The primary outcome was post-treatment patient functioning. Results: The CBT+TAU group showed a greater improvement in functioning, which was measured using the Global Assessment Functioning (GAF) and Functioning Assessment Short Test (FAST), compared to the TAU group post-treatment. The CBT+TAU participants exhibited a greater decline in depressive, negative, and general psychotic symptoms; a better awareness of the disease and treatment adherence; and a greater increase in brain-derived neurotrophic factor levels than TAU participants. Conclusions: Early intervention based on a combined clinical treatment involving CBT as an adjunctive to standard treatment may improve clinical and functional outcomes in FEP.

## 1. Introduction

First-episode psychosis (FEP) is characterised by relapses, especially if not adequately treated. This may have a negative impact on clinical and functional outcomes in the long term [1]. Factors that have been associated with poorer outcomes include a poor premorbid adjustment, comorbid substance use disorders, greater severity of negative symptoms, history of suicide attempts and suicidal ideation, longer duration of untreated psychosis (DUP), and lack of treatment adherence [2]. Brain-derived neurotrophic factor (BDNF) is a neurotrophin that plays a critical role in neurodevelopment and brain plasticity, regulating the function, survival, repair, and differentiation of neurons [3]. Moreover, BDNF has been suggested to be a useful neurobiological biomarker of early-onset schizophrenia [4,5,6,7,8,9,10]. A systematic review and meta-analysis conducted by Sanada et al. [7] revealed that the effect of psychological and non-pharmacological treatments on BDNF levels has been scarcely studied; however, according to the existing literature, they could potentially have an influence on this biological parameter.

The first five years after the onset of the illness, i.e., ‘the critical period’, might be an indicator of long-term prognosis and is when FEP patients are more likely to relapse. Patients in this period are also more responsive to treatment than in later stages of the illness [11]. Early treatment of FEP during this period is crucial to prevent relapses and reduce disabilities caused by the disease. There is convincing evidence that early intervention may contribute to improvement in the prognosis and course of FEP. Early intervention is focused on increasing treatment adherence and disease awareness, improving symptomatic and functional outcomes, and reducing relapses and hospitalisations [12]. Clinical practice guidelines for the early treatment of psychosis recommend an integrated approach based on evidence-based psychosocial treatments, such as cognitive behavioural therapy (CBT), family therapy, and/or psychoeducation, as an adjunctive to pharmacological treatment (13–19). These early intervention programmes for psychosis have demonstrated feasibility and effectiveness in improving symptomatic and functional outcomes in FEP. Patients treated during the earliest stages of psychosis experienced fewer symptoms, relapses, and disability and showed a better functioning and quality of life, compared to patients who received treatment-as-usual (TAU) [13,14,15,16,17,18,19,20,21,22,23,24,25,26,27,28]. While evidence of psychosocial interventions for FEP has shown them to be effective in improving symptoms and functionality, additional high-quality randomised clinical trials (RCTs) with representative samples of FEP patients that allow for generalisation of the results are needed. A larger number of trials of a good methodological quality are also required to determine the most effective psychological treatments for FEP patients.

Accordingly, we conducted a RCT to compare the efficacy of a combined clinical treatment for FEP involving a CBT adjunctive to TAU (CBT+TAU) versus TAU alone. The data were compared at baseline and post-treatment. The primary objective of this study was to assess the improvements in patient functioning. The secondary objectives were: (1) to compare the efficacy of CBT+TAU versus TAU in improving psychotic and mood (anxiety and depressive) symptoms; (2) to compare the treatment adherence and awareness of disease in CBT+TAU vs. TAU; and (3) to compare the BDNF levels between the groups.

## 2. Materials and Methods

### 2.1. Participants

**Figure 1:**
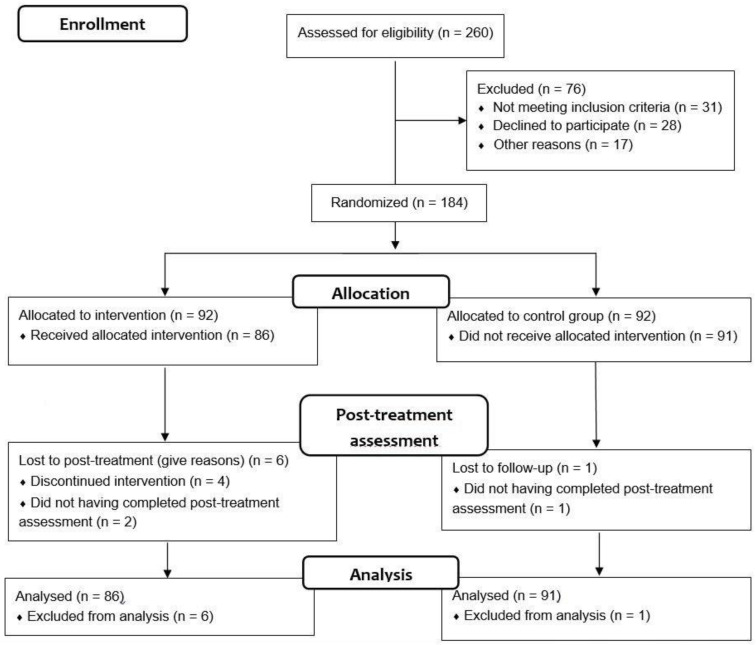
The CONSORT flow diagram.

This was a multicentre, single-blind RCT registered in 2013 (NCT01783457). The study protocol was described in a previous article by Barbeito et al. [29]. This RCT complied with the CONSORT (CONsolidated Standards of Reporting Trials) guidelines, checklist, and flow diagram (Figure 1).

All procedures contributing to this work complied with the ethical standards of the relevant national and institutional ethics committees for human experimentation and were performed in accordance with the principles of the Helsinki Declaration of 1975, revised in 2008. The study was approved by the Clinical Research Ethics Committees of all participating centres: Araba University Hospital: HS/PI2010009, Clinic Hospital of Barcelona: 2010/5890, Clinic University Hospital of Valencia: 2010/2904, University Hospital of Bellvitge: PR079/10, and the University of Valencia: 2010/2904.

The recruitment of FEP patients was conducted between January 2011 and June 2015. After a comprehensive literature review, the required sample size was estimated using the Ene 2.0 software package. Based on similar previous studies [26,30], to achieve a 90% power to detect differences of 6 points or more between groups in the mean of the primary outcome (Global Assessment of Functioning, GAF) [31], and assuming an alpha risk of 0.05, 130 patients were needed for each group.

The patients needed to be between 18–45 years old and to have received a diagnosis of FEP, according to the 4th edition text-revised of the Diagnostic and Statistical Manual of Mental Disorders (DSM-IV-TR) [32], within the previous five years, which could include either schizophreniform disorder, schizoaffective disorder, brief psychotic disorder, delusional disorder, non-specified psychotic disorder, bipolar disorder with psychotic symptoms, or major depressive disorder with psychotic symptoms. The exclusion criteria were intellectual disability, organic brain disorders or the presence of comorbidities that could hinder communication, and substance use disorder as the primary diagnosis.

### 2.2. Instruments

Sociodemographic (age, sex, level of education, and living and employment status) and clinical (detailed below) data were collected using a clinical interview at baseline. The clinical variables were also gathered during the post-treatment assessment.

#### 2.2.1. Primary Outcome

The primary outcome was the level of post-treatment functioning, as measured by the Global Assessment of Functioning (GAF) scale [31], which evaluates general activity and functioning, and the Functioning Assessment Short Test (FAST) [33]. These were used to assess six areas of functioning: autonomy, occupational functioning, cognitive functioning, financial issues, interpersonal relationships, and leisure time. This scale has shown strong psychometric properties in Spanish individuals with FEP [34].

#### 2.2.2. Secondary Outcomes

Clinical variables, including clinical global impression, psychosis, anxiety and depressive symptoms, treatment adherence, and awareness of disease assessed at baseline and at post-treatment, were evaluated using an extensive battery of instruments.

The patients were diagnosed according to the DSM-IV-TR criteria using the Structured Clinical Interview for the Diagnostic and Statistical Manual of Mental Disorders, Axis I Disorders (SCID-I) [35].

The Clinical Global Impression Scale (CGI) [36] was used to measure the severity and improvement in global symptoms. CGI has two components: CGI-Severity (CGI-S), which rates illness severity, and CGI-Improvement (CGI-I), which rates changes from the initiation of treatment.

Positive, negative, and general psychotic symptoms were assessed using the Positive and Negative Syndrome Scale (PANSS) [37,38].

In order to measure the anxiety experienced by the subject in the previous week (anxiety-state) and usual reactions to certain situations (anxiety-trait), the State-Trait Anxiety Inventory (STAI) [39,40] was used.

Depressive symptomatology was assessed with the Hamilton Rating Scale for Depression (HRSD) [41,42].

Patient disease awareness was quantified using the Scale Unawareness of Mental Disorder (SUMD) [43,44], and the 4-item Morisky Medication Adherence Scale (MMAS) [45,46] was used to explore the attitudes of patients towards their treatment, categorising patients according to a good or bad adherence.

Plasma BDNF levels were analysed using a BDNF Sandwich enzyme-linked immunosorbent assay (ELISA) kit (CYT306; EMD Millipore Corporation, Billerica, MA, USA), according to the manufacturer’s instructions. Blood cells were immediately separated from plasma and processed. Standard curves were constructed using plasma duplicates. The absorption at 450 nm was measured with a Synergy HT microplate reader (BioTek Instruments, Winooski, VT, USA). The minimum detection limit was 7.8 pg/mL, and the assay range was from 7.8 to 500 pg/mL. The intra- and inter-assay coefficients of variation for the BDNF ELISA kits were +3.7% and +8.5%, respectively.

### 2.3. Procedure

The patients were recruited from different assistance levels of psychiatric care (including the outpatient clinic, day hospital, and inpatient unit) of five centres associated with the Center for Biomedical Research in the Mental Health Network (CIBERSAM): Araba University Hospital, Hospital Clinic of Barcelona, Clinic University Hospital of Valencia, University Hospital of Bellvitge, and University of Valencia. The patients needed to be psychopathologically stable prior to the start of the psychological intervention; therefore, the treatment was administered after discharge.

After signing informed consent, the patients were randomised to one of the treatment groups (TAU or combined clinical treatment) using random allocation software. The allocation sequence was designed by an independent person not otherwise involved in the trial.

The patients were evaluated at baseline and at post-treatment (which corresponds to the end of the psychological treatment in the case of the intervention group). At these two time points, plasma samples were collected to determine BDNF levels. The subjects of both treatment groups were assessed by an evaluator who was not the therapist who developed the intervention. The assessment was thus carried out by a researcher who was blind to the patient allocation process. The evaluators of all the centres were trained to use scales for inter-rater reliability by rating each of the scales with practical cases. For each scale, the inter-rater reliability was higher than or close to 0.8, thus ensuring the inter-rate reliability of patient assessments: (SCID-I (kappa = 0.88; CGI (kappa = 0.94), PANSS, kappa = 0.80; STAI, kappa = 0.77; GAF, kappa = 0.95; HDRS, kappa = 0.79; and SUMD, kappa = 0.81; MMAS, kappa = 0.94, FAST, kappa = 0.88). The therapists from all centres received an 8-h face-to-face training session and were provided with the same materials that would later be offered to the patients in the intervention programme. To further enhance the reliability, doubts that arose during the treatment administration were addressed by the coordinating centre via video conferencing (Web Ex Training CENTER). TAU was provided in all centres, according to the standard procedures of the Spanish National Health Service for FEP patients. In the two treatment groups, medication was allowed to be adjusted when necessary by the assigned psychiatrist in order to guarantee participant welfare, according to ethical standards.

### 2.4. Intervention Programme

The patients were randomly assigned to one of the treatment groups (either TAU or combined clinical treatment).

TAU refers to the standard treatment provided to FEP patients. This includes pharmacological treatment and regular sessions with the assigned psychiatrist and a multidisciplinary team in each centre. TAU includes physical care, career counselling, and the provision of unstructured information to families about disease symptoms, treatment, and prognosis.

The combined clinical treatment involved TAU plus an adjuvant individual CBT intervention. The intervention programme was implemented in all participating centres, addressing the same content and following the same structure. It was composed of 14 one-hour sessions fortnightly (±3 days) for a period of 6.5–7.5 months. The first part of the program (sessions 1–9) was composed of psychoeducational sessions aimed at improving patients’ insight into their illness, treatment adherence, prodromal identification, early intervention to prevent relapses, and a healthy lifestyle. The second part of the intervention (sessions 10–14) included cognitive behavioural techniques for symptom and thought management (anxiety management techniques and social and problem-solving skills). The patients were offered the possibility of using a telephone helpline between sessions if they had any questions related to the content of the sessions or their status. The program included the following sessions:

1. What is a first episode of psychosis?
2. Challenge and importance of insight into vulnerability.
3. Symptom recognition.
4. Prevention of relapses: protective and risk factors.
5. Detection of prodromes.
6. What can I do if I perceive that the symptoms are emerging again?
7. Treatment adherence.
8. Healthy lifestyles: sleep and sexuality.
9. Healthy lifestyles: substance use.
10. Anxiety management techniques (I).
11. Anxiety management techniques (II).
12. Social skills: assertiveness techniques.
13. Problem-solving techniques.
14. Final doubts and farewell.

### 2.5. Statistical Analyses

All statistical analyses were performed using SPSS Statistics for Windows (Windows v23.0), with the significance level set at *p* ≤ 0.05.

The baseline sociodemographic and clinical characteristics of the IT and the TAU groups were compared using *χ*^2^-tests for categorical variables and Student’s *t*-test for independent samples for quantitative variables.

Within-group changes (pre-post treatment) in symptomatology, functioning, and BDNF levels were examined with Student’s *t*-test for paired samples. McNemar tests for dichotomous variables were used to analyse changes in adherence.

An analysis of covariance (ANCOVA) was performed in order to determine differences between groups in terms of clinical mean changes between the baseline and post-treatment. The baseline values were used as covariates to eliminate the possible influence of initial score variances on the outcomes. The partial eta squared (${ƞ}_{p}^{2}$) was also calculated to assess the effect size. Since adherence was assessed using dichotomous scores, the differences were evaluated using logistic regression models adjusted for baseline adherence.

Finally, we analysed the influence of clinical improvements in the functional outcomes of each group. First, a simple linear regression was performed, using the GAF post-treatment score as the dependent variable and post-treatment clinical variables as independent variables. The variables found to be significant in the univariate regression were entered into the final multivariate model.

## 3. Results

### 3.1. Sociodemographic and Clinical Baseline Characteristics of the Sample

The initial sample was composed of 184 FEP patients (92 for each intervention group). Of these subjects, 7 (1 patient of the TAU group and 6 patients of the CBT+TAU group) were excluded from the analysis for not having attended all intervention sessions or the post-treatment assessment. As a result, the final sample consisted of 177 patients, of whom 91 (51.4%) were assigned to the TAU group and 86 (48.6%) to the CBT+TAU group (Figure 1). A total of 105 (59.3%) subjects were male, and 72 (40.7%) were female. Additionally, 67.9% (39.2% and 28.7%, respectively) were employed and/or students. Most of the patients (141) (82.5%) were single, and 106 (72.1%) had completed secondary education. The diagnoses were similarly distributed in both groups, with the most frequent diagnosis being non-specified psychotic disorder (*χ*^2^ = 2.500; *p* = 0.776). The majority of patients were treated with second-generation antipsychotics (97.2%), polytherapy (79.8%), and monotherapy (20.2%). There were no clinical or functional differences at baseline between the CBT+TAU and the TAU groups. There were also no differences between the groups regarding BDNF levels (*t* = −0.839; *p* = 0.404).

The baseline sociodemographic and clinical characteristics of each group are represented in Table 1.

### 3.2. Clinical and Functional Outcomes of FEP Patients

Regarding the within-group mean changes, there was a significant improvement between the baseline and post-treatment, except for CGI-S, both in the CBT+TAU (*t* = 1.519; *p* = 0.133) and the TAU groups (*t* = 0.380; *p* = 0.705). There were no changes in BDNF levels between the baseline and post-treatment in any of the groups (Table 2).

In relation to the progression (baseline/post-treatment) of the CBT+TAU and TAU, significant differences were found in clinical outcomes (Table 3). For the primary outcome, the CBT+TAU group showed a greater improvement in functioning, as assessed by GAF (F = 6.269; *p* = 0.013, ${ƞ}_{p}^{2}=0$.04) and FAST (F = 5.468; *p* = 0.021, ${ƞ}_{p}^{2}=0$.04). Regarding secondary outcomes, the CBT+TAU T showed a greater decline in positive (PANSS P) (F = 6.214; *p* = 0.014, ${ƞ}_{p}^{2}=0$.04), negative (PANSS N) (F = 4.008; *p* = 0.047, ${ƞ}_{p}^{2}=0$.03), and general (PANSS G) (F = 4.626; *p* = 0.033, ${ƞ}_{p}^{2}=0$.03) psychotic symptoms, compared to the TAU group. The CBT+TAU group also had a greater reduction in depressive symptoms (HDRS) (F = 4.078; *p* = 0.045, ${ƞ}_{p}^{2}=0$.03) and better disease awareness (SUMD) (F = 6.564; *p* = 0.011, ${ƞ}_{p}^{2}=0$.04), compared to the TAU group. In addition, the percentage of patients with a good adherence increased significantly in the CBT+TAU group (12.4% vs. 1.9%; B = 1.002, *p* = 0.007, OR = 2.723). There was also an increase in BDNF levels in the CBT+TAU group, compared to the TAU group (F = 3.923; *p* = 0.050, ${ƞ}_{p}^{2}=0$.05). The effect sizes for all these clinical outcome differences were medium and high in terms of adherence outcomes, with an OR = 2.723.

### 3.3. Influence of Clinical Improvements on Functional Outcomes

In the CBT+TAU group, all the post-treatment clinical variables had an impact on the functional outcomes, whereas in the TAU group, the HDRS (*B* = −0.859; 95% CI: −1.216 to −0.503; *p* ≤ 0.001) and STAI-S (*B* = −0.563; 95% CI: 72.742 to 90.601; *p* = 0.006) scores were associated with a poorer functioning. When significant variables in the simple linear regression were included in the multivariate model, the results indicated that improvements in the CGI-I (*B* = −0.693; 95% CI: −0.966 to −0.420; *p* ≤ 0.001), PANNS-N (*B* = −0.647; 95% CI: −0.985 to −0.309; *p* ≤ 0.001), and STAI-S (*B* = −0.232; 95% CI: −0.455 to −0.010) post-treatment scores were associated with a better functioning in the CBT+TAU group, whereas in the TAU group, only the HDRS (*B* = −2.091; 95% CI: −2.635 to −1.548; *p =* 0.000) was associated with functioning.

## 4. Discussion

This randomised controlled trial aimed to compare the efficacy of a CBT+TAU treatment of FEP versus TAU alone, comparing the results at baseline with post-treatment outcomes. The patients in the intervention group exhibited a more pronounced improvement in functioning, treatment adherence, and awareness of their disease, as well as a greater reduction in depressive, negative, and general psychotic symptoms post-treatment, as compared to TAU. Additionally, the BDNF levels were higher in the intervention group than in the TAU group. Our findings support the importance of early psychological intervention, as an adjunctive to pharmacological treatment, for clinical and functional improvements in FEP. In a recent systematic review, meta-analysis, and meta-regression, Correll et al. [21] analysed 10 RCTs and concluded that early intervention programmes were associated with better clinical and functional outcomes than TAU at post-treatment, including hospitalisation risk, bed days, the severity of symptoms, and global functioning.

The primary outcome of the study is that patients who received CBT+TAU, in consonance with other studies [20,21,22,23,24,25,26,27,28], had better functional outcomes than those who only received TAU. Notably, improvements in clinical symptomatology had an influence on functional outcomes. Specifically, improvements in the severity of symptoms and anxious and negative psychotic symptoms were associated with better functioning in the intervention group, whereas in the TAU group, only depressive symptoms were associated with poor functioning. A functional impairment is present in early psychosis and, although the symptoms of FEP generally improve in the short term, patients often develop poor functionality during post-treatment follow-up and even 12 months after early intervention [47]. Therefore, interventions targeting functional recovery in FEP are necessary, and our results show that psychoeducational intervention could be useful in improving clinical symptoms, which influences functional outcomes in these patients.

One of the main findings of our study is that the BDNF levels were improved in the CBT+TAU group, compared to TAU at post-treatment. This suggests that BDNF levels are reduced at the onset of psychosis and that a psychological intervention could restore the plasma concentrations of this neurotrophic factor. These results are consistent with several clinical studies reporting reduced BDNF levels in FEP, which might be a marker of early-onset schizophrenia. Inflammatory processes, neurotrophin levels, and functional status seem to be related to disease onset [4,5,6,7,8,9,10]. Specifically, BDNF receptor expression is associated with the treatment response and overall functioning at disease onset and after one-year follow-up [6].

The second main finding of the study is that, in agreement with other studies [23,25], the intervention was effective in reducing psychotic symptoms. The psychological treatment had a remarkable impact on negative symptoms, which are usually associated with a worse response to pharmacological treatment in FEP [48,49]. Moreover, the persistence of negative symptoms at follow-up leads to a worse functioning in patients [50], which highlights the significant role of negative symptoms in early interventions. Other studies also concluded that specific psychological interventions are useful in ameliorating negative symptoms in early psychosis [25,51].

Our psychological intervention also proved, as in other studies [17], to be effective in improving depressive symptoms. Depressive symptoms are common in FEP and often persist over the course of the disease. In a recent systematic review and meta-analysis conducted by McGinty and Upthegrove [52], depressive symptoms in FEP were associated with a poorer long-term functioning and a reduced chance of achieving functional remission. In a previous study, we reported that subclinical depressive symptoms were also associated with cannabis abuse, which could be a predictor of negative outcomes in FEP [53]. Hence, depressive symptoms should be a therapeutic target in FEP patients to prevent the development of an unfavourable clinical and functional disease course.

The third finding of the study is that psychoeducational intervention was effective in improving adherence and disease awareness in FEP. A lack of treatment adherence is associated with poor clinical and functional outcomes. Moreover, a high number of FEP patients are non-adherent to treatment at 12-month follow-up, which increases the risk of relapse and rehospitalisations [2,11,54,55]. Our results, according to other studies, revealed that psychoeducational intervention could be crucial for improving adherence to medication and illness course, and that clinical outcomes of patients not only depend on initial treatment but also on continued adherence to treatment. The study conducted by Randall et al. [56] found that the Early Psychosis Prevention and Intervention Service programme was associated with an increased adherence to antipsychotic medication use during and after the program, thus improving patient conditions. Early intervention in FEP must include psychosocial interventions focused on increasing adherence and improving therapeutic alliance. A lack of insight, negative attitudes toward medication, and a bad therapeutic alliance are factors associated with non-adherence [57] and which could be targeted by such interventions to achieve better outcomes in patients with FEP.

In conclusion, the results of this study suggest that early interventions in FEP based on a combined clinical treatment involving a CBT adjunctive to TAU may help improve clinical and functional outcomes, in addition to restoring the BDNF levels of FEP patients. Hence, treating FEP with a standard treatment alone may not be effective enough for the majority of patients, and an integrative approach is necessary to optimise the functional outcomes [58].

This study has some limitations. First, long-term follow-up should be considered in future research to determine long-term benefits and assess other aspects, such as relapses or rehospitalisations. Secondly, it should be considered that the components and number of sessions of different psychological intervention programmes may vary between studies. Third, our psychological treatment programme has an individual format, while others employ a group approach. Moreover, in our study, although all the centres included are public and belong to the Spanish National Health Service, with a set time of 30 min for each visit, there are some slight differences between the centres. Additionally, the differences in the time and attention devoted to each treatment arm should be taken into account in the interpretation of the results. A little more time was spent with patients in the enriched treatment group than with the control group. According to ethical requirements, the TAU group received the psychological intervention after the completion of the study. Another limitation is that, although minimal medication dose adjustments were allowed during the trial, this variable might have an influence on the study outcomes. Finally, because the pre-calculated size was not reached, the final size of the groups was reduced. As this could affect the pre-calculated power of the study, we corrected the statistical calculation, and the final power of the study was set to 80%.

The study also has important strengths, which give our findings relevant implications for clinical practice. CBT+TAU has proven to be a useful therapeutic approach to treat and achieve recovery in early psychosis. Moreover, the results are generalisable, because they were obtained in a multicentre study that included a large sample of patients recruited in different Spanish psychiatric admission centres for acute psychosis.

## Figures and Tables

**Table 1:** Baseline sociodemographic and clinical characteristics of the sample.

Variable		Total (*n* = 177)	CBT + TAU	TAU	*t/χ* ^2^	*p*
Sex	Women	72 (40.7%)	39 (45.3%)	33 (36.3%)	*χ*^2^ = 1.512	0.219
Age		28.53 ± 8.97	27.74 ± 7.66	29.27 ± 10.03	*t* = −1.136	0.258
Occupation	Employed	69 (39.2%)	31 (35.7%)	39 (42.5%)	*χ*^2^ = 0.863	
	Unemployed	57 (32.2%)	30 (34.5%)	27 (29.9%)		0.650
	Student	51 (28.7%)	26 (29.8%)	25 (27.6%)		
Education level	Primary school	6 (3.4%)	1 (1.4%)	5 (5.3%)	*χ*^2^ = 2.784	0.249
	Secondary school	128 (72.1%)	67 (77.5%)	61 (67.1%)		
	College	43 (24.5%)	18 (21.1%)	25 (27.6%)		
Civil status	Single	146 (82.5%)	72 (83.3%)	74 (81.6%)	*χ*^2^ = 0.136	
	Married	23 (12.9%)	10 (11.9%)	13 (13.8%)		0.934
	Divorced	8 (4.7%)	4 (4.8%)	4 (4.6%)		
Diagnosis	Non-specified psychotic disorder	118 (66.7%)	58 (67.4%)	60 (65.9%)	*χ*^2^ = 2.500	0.776
	Bipolar disorder with psychotic symptoms	23 (13%)	13 (15.1%)	10 (11%)		
	Schizophreniform disorder	17 (9.6%)	6 (7%)	11 (12.1%)		
	Brief psychotic disorder	13 (7.3%)	6 (7%)	7 (7.7%)		
	Major depressive disorder with psychotic symptoms	3 (1.7%)	1 (1.2%)	2 (2.2%)		
	Delusional disorder	3 (1.7%)	2 (2.3%)	1 (1.1%)		
Treatment	Antipsychotics	171 (97.2%	85 (98.8%)	86 (95.6%)	*χ*^2^ = 1.716	0.190
	Benzodiazepines	77 (43.8%9	35 (40.7%)	42 (46.7%)	*χ*^2^ = 0.637	0.425
	Mood stabilisers	24 (13.6%)	12 (14%)	12 (13.3%)	*χ*^2^ = 0.014	0.905
	Antidepressants	20 (11.4%)	11 (12.8%)	9 (10%)	*χ*^2^ = 0.340	0.560
FAST		31.61 ± 15.81	33,09 ± 15,519	30,16 ± 16,053	*t* = −1.218	0.225
GAF		58.54 ± 11.0	59.63 ± 9.99	56.78 ± 11.82	*t* = 1.133	0.259
CGI-S		7.64 ± 5.75	7.74 ± 5.94	7.55 ± 5.60	*t* = 0.224	0.823
CGI-I		8.65 ± 7.77	9.06 ± 8.10	8.26 ± 7.46	*t* = 0.677	0.499
PANSS P		14.16 ± 6.95	13.40 ± 6.46	15.36 ± 7.71	*t* = −1.960	0.052
PANSS N		13.69 ± 6.27	14.48 ± 6.96	13.19 ± 5.83	*t* = 1.635	0.104
PANSS PG		29.19 ± 7.98	29.64 ± 8.68	28.77 ± 7.2	*t* = 0.886	0.377
HDRS		11.75 ± 7.40	12.46 ± 8.22	11.07 ± 6.51	*t* = 1.241	0.219
STAI-State		24.82 ± 10.90	23.87 ± 10.96	26.14 ± 10.59	*t* = −0.892	0.374
SUMD		4.94 ± 3.18	5.50 ± 3.05	5.47 ± 3.95	*t* = −0.054	0.957
MMAS		97 (54.8%)	52 (60.0%)	45 (49.4%)	*χ*^2^ = 1.940	0.164
BDNF (pg/Ml)		6.95 ± 5.77	7.66 ± 6.79	6.49 ± 5.05	*t* = −0.839	0.404

**Table 2:** Within-group changes (pre/post-treatment).

Group	CBT + TAU			TAU		
Variable	Pre	Post	*t (p)*	Pre	Post	*t (p)*
FAST	33.43 ± 15.531	15.15 ± 16.425	7.638 (≤0.001)	30.96 ± 20.05	20.05 ± 15.221	6.109 (≤0.001)
GAF	61.24 ± 10.62	76.03 ± 13.60	−7.669 (≤0.001)	56.92 ± 12.03	69.58 ± 13.91	−7.310 (0.001)
CGI-S	6.74 ± 5.43	6.09 ± 6.61	1.519 (0.133)	7.16 ± 5.41	6.99 ± 6.68	0.380 (0.705)
CGI-I	8.08 ± 7.81	7.07 ± 7.57	2.131 (0.037)	7.48 ± 7.03	6.25 ± 6.09	3.033 (0.003)
Panns P	13.34 ± 6.44	8.47 ± 3.22	6.365 (≤0.001)	15.39 ± 7.66	10.39 ± 4.90	5.705 (≤0.01)
Panns N	14.78 ± 7.49	10.55 ± 5.71	5.394 (≤0.001)	13.41 ± 6.29	11.39 ± 5.43	3.787 (≤0.001)
Panns G	30.17 ± 10.33	21.35 ± 7.60	6.738 (≤0.001)	28.81 ± 7.29	23.33 ± 6.67	6.861 (≤0.001)
HDRS	12.31 ± 8.31	5.24 ± 5.58	6.434 (≤0.001)	11.30 ± 6.28	6.65 ± 4.96	8.066 (≤0.001)
STAI-State	24.11 ± 10.91	19.05 ± 9.18	3.192 (0.002)	25.97 ± 11.30	21.28 ± 7.93	3.309 (0.002)
SUMD	4.67 ± 3.05	3.07 ± 2.88	6.067 (≤0.001)	4.64 ± 2.97	3.93 ± 3.03	2.697 (0.008)
MMAS (good) ^1^	51 (60.0%)	55 (72.4%)	*p* = 0.052 ^1^	38 (43.7%)	36 (45.6%)	*p* = 0.690 ^1^
BDNF (pg/Ml)	7.66 ± 6.79	9.76 ± 6.78	−1.760; (0.090)	6.49 ± 5005	6.95 ± 4.39	−0.675 (0.503)

^1^ *p* value obtained with the Mcnemar test for dichotomous variables.

**Table 3:** A comparison of the course of the disease, adjusting for pre-treatment scores (ANCOVA).

Variable	CBT + TAU Δ	TAU Δ	*F*	*p*	${ƞ}_{p}^{2}$
FAST	−18.28 ± 20.73	−10.91 ± 15.88	5.468	0.021	0.04
GAF	14.79 ± 16.70	12.66 ± 15.78	6.269	0.013	0.04
CGI-S	−0.65 ± 3.66	−0.17 ± 3.98	0.605	0.438	0.00
CGI-I	−1.01 ± 3.98	−1.24 ± 3.62	0.379	0.539	0.00
Panns P	−4.87 ± 6.67	−5.00 ± 7.94	6.214	0.014	0.04
Panns N	−4.22 ± 6.83	−2.02 ± 4.84	4.008	0.047	0.03
Panns G	−8.83 ± 11.34	−5.48 ± 7.19	4.626	0.033	0.03
HDRS	−7.07 ± 9.51	−4.65 ± 5.16	4.078	0.045	0.03
STAI State	−5.05 ± 11.95	−4.69 ± 11.69	1.494	0.224	0.01
SUMD	−1.60 ± 2.28	−0.71 ± 2.43	6.564	0.011	0.04
MMAS (good) ^1^	12.4%	1.9%	1.002	0.007	2.723
BDNF (pg/Ml)	2.10 ± 6.30	0.46 ± 4.55	3.923	0.050	0.05

^1^ Variable analysed by logistic regression, adjusted for pre-treatment adherence. The data are represented as the B coefficient, *p*-value, and odds ratio.

## Data Availability

The data are contained within the article.
